# Use of a highly-sensitive rapid diagnostic test to screen for malaria in pregnancy in Indonesia

**DOI:** 10.1186/s12936-020-3110-6

**Published:** 2020-01-16

**Authors:** Vera T. Unwin, Rukhsana Ahmed, Rintis Noviyanti, Agatha M. Puspitasari, Retno A. S. Utami, Leily Trianty, Theda Lukito, Din Syafruddin, Jeanne R. Poespoprodjo, Maria A. Santana-Morales, Feiko O. Ter Kuile, Emily R. Adams

**Affiliations:** 1 Department of Tropical Disease Biology and Department of Clinical Sciences, Liverpool School of Tropical Medicine, Liverpool, L3 5QA UK; 2 Malaria Pathogenesis Laboratory, Eijkman Institute of Molecular Biology, Jakarta, Indonesia; 3 Clinesia, Jakarta, Indonesia; 4 Mimika District Health Authority, District Government Building, Jl. Cendrawasih, Timika, 99910 Papua Indonesia; 5 Timika Malaria Research Programme, Papuan Health and Community Development Foundation, Jl. SP2-SP5, RSMM Area, Timika, 99910 Papua Indonesia; 6 Department of Child Health, Faculty of Medicine, University Gadjah Mada, Jl. Kesehatan no 1, Sekip, Yogyakarta, 55284 Indonesia; 7 University Institute of Tropical Diseases and Public Health of the Canary Islands, University of la Laguna, Tenerife, Spain; 8 Network Biomedical Research on Tropical Diseases, RICET, Madrid, Spain

**Keywords:** Malaria, Diagnostics, Pregnancy, Rapid diagnostic test, Diagnostic performance, Molecular techniques

## Abstract

**Background:**

The sensitivity of rapid diagnostic tests (RDTs) for malaria is inadequate for detecting low-density, often asymptomatic infections, such as those that can occur when screening pregnant women for malaria. The performance of the Alere™ Ultra-sensitive Malaria Ag *Plasmodium falciparum* RDT (uRDT) was assessed retrospectively in pregnant women in Indonesia.

**Methods:**

The diagnostic performance of the uRDT and the CareStart™ Malaria HRP2/pLDH VOM (*Plasmodium vivax, Plasmodium ovale* and *Plasmodium malariae*) Combo RDT (csRDT) were assessed using 270 stored red blood cell pellets and plasma samples from asymptomatic pregnant women. These included 112 *P. falciparum* negative and 158 *P. falciparum* positive samples detected by a composite test (qPCR, LAMP, nPCR) as reference standard. Diagnostic indicators: sensitivity, specificity, negative predictive value (NPV), positive predictive value (PPV), diagnostic odds ratio (DOR) and the level of agreement (kappa) were calculated for comparison.

**Results:**

Compared with the reference test, the uRDT had a sensitivity of 19.6% (95% CI 13.9–26.8) and specificity of 98.2% (93.1–99.7%). The csRDT was 22.8% (16.7–30.3) sensitive and 95.5% (89.4–98.3) specific for *P. falciparum* infections. Performance of the uRDT was non-significantly different to the csRDT (p = 0.169). RDT outcome was stratified by qPCR cycling threshold (Ct), and performance of the RDTs was found to be comparable across parasite loads.

**Conclusion:**

The uRDT performed similarly to the currently used csRDTs in detecting *P. falciparum* infections in asymptomatic pregnant women. In these settings, molecular diagnostics are currently the most sensitive for malaria.

## Background

Control programmes for malaria will need to account for the changing epidemiology of malaria as countries progress toward elimination. The current goal to eliminate malaria in 31 countries by 2030 will require sensitive diagnostic tools [1] to detect the reservoir of low-density and often sub-patent infections [2–5]. Although the infectivity of these asymptomatic individuals is low, modelling shows that they contribute significantly to onward transmission because they make up the majority of the population in low-transmission areas [4, 6].

Diagnosis of malaria during pregnancy is complicated by the ability of certain *Plasmodium falciparum* parasites to sequester in the placenta, which can result in parasite densities in the peripheral blood below the level of detection of existing Rapid Diagnostic Tests (RDTs) and light microscopy [7–11]. Diagnosis and treatment of these infections may improve pregnancy outcomes [12, 13] by preventing them from developing into patent infections and may control transmission by reducing the parasite reservoir [3, 8, 12, 13]. Lateral-flow rapid diagnostic tests detecting circulating malaria antigen are simple, widely used, and the cheapest point-of-care diagnostic tool for malaria. Many countries in the Asia–Pacific region use “Test and Treat” strategies at the first antenatal booking visit to screen all pregnant women for malaria regardless of symptoms [14–16], or are exploring intermittent screening and treatment (IST) strategies to test women at each scheduled antenatal visit [13, 17]. However, the current generation of standard RDTs for malaria has a limit of detection of 100 parasites/µl and is not designed to detect low-density infections in asymptomatic pregnant women [2]. Low-cost and field-deployable highly sensitive rapid diagnostics may improve the detection of malaria in these populations. [2, 18].

Currently, only nucleic acid amplification tests (NAATs) are sufficiently sensitive to detect these low-density infections [2]. However, these methods are limited to well-equipped laboratory settings due to their inherent complexity and need for laboratory equipment.

Recently, the new Alere™ Ultra-sensitive Malaria Ag *P. falciparum* RDT (uRDT) was developed to bridge the gap between high-sensitivity and field-ready diagnostics [19]. To date, studies in Uganda, Ethiopia, Myanmar and Papua New Guinea have shown superior sensitivity of the uRDT in comparison to widely used SD Bioline RDTs in asymptomatic general population. An additional laboratory study reports a log-fold lower limit of detection (LOD) for the HRP2 antigen (80 pg/ml) [19]. The only study in pregnant women did not observe notable differences in sensitivity between the uRDT and existing SD Bioline RDTs. [20].

This study describes the performance of the uRDT and standard RDT CareStart™ Malaria HRP2/pLDH *Pf*/VOM Combo (csRDT) in stored blood specimens of asymptomatic pregnant women in Indonesia compared to a composite molecular tests.

## Methods

### Study sites and sample collection

The study was performed using stored samples of red blood cell pellets (pRBC) reconstituted with plasma collected during a recently completed STOPMIP trial (ISRCTN: 34010937) comparing intermittent preventive treatment or intermittent screening and treatment strategies with single screen and treatment in pregnant women in Indonesia [17]. The trial was conducted between 2013 and 2016 in Timika in Mimika District southern Papua-Indonesia, an area with moderate, year-round malaria transmission [21]. All four major species of *Plasmodium* are endemic in the area with *P. falciparum* accounting for 57.9% of infections, *Plasmodium vivax*—33.8%, *Plasmodium malariae*—4.4% and *Plasmodium ovale*—0.5% amongst pregnant women [21]. Co-infections of *P. falciparum* and *P. vivax* account for the remaining 3.8%.

Whole blood samples were taken from asymptomatic pregnant women (> 16 weeks gestation) at antenatal visits to screen for malaria infection. During the trial this included microscopy, molecular malaria diagnostics (qPCR), nested PCR (nPCR), and loop-mediated isothermal amplification (LAMP). Malaria RDTs (First Response Malaria Ag pLDH–HRP2 Combo [I16FRC30]; Premier Medical Corporation, Nani Daman, India) were also done at enrolment in the single-screen and treat and intermittent screen and treat arms of the STOPMIP trial. The FirstResponse RDT data is not included in this study as the number of matched samples was low.

### Sample processing

During STOPMIP trial, venous blood (4 ml) was collected from each participant in EDTA tubes at the booking and delivery visits and processed within 24 h. Samples were centrifuged for 10 min at 15,000 rpm and RBCs and plasma separated and stored at − 20 °C. Dried blood spots (DBS) were also prepared at the field laboratory, where 10 µl blood was pipetted on to filter paper (Whatman 3MM) and left to air-dry, stored in zip-lock bags and transported to the Eijkman institute of molecular biology for molecular testing.

### RDTs

The RDTs used here recommend using whole blood samples. Due to storage conditions in the trial pellets of frozen RBCs were first reconstituted by adding the corresponding plasma sample to a final haematocrit (HCT) of 30%. This reflects the average haemoglobin level (~ 11 g/dL) observed previously in this population [18, 22] and in STOPMiP participants [17]. The Alere™ Ultra-sensitive Malaria Ag *P. falciparum* RDT (uRDT, Standard Diagnostic Inc. Suwon City, South Korea, Catalogue No: 05FK140, Lot No. 05LDC002A) detects *P. falciparum* infections only. The CareStart RDT (csRDT) (CareStart™ Malaria HRP2/pLDH Pf/VOM Combo RDT, Catalogue No: G0171, Lot No. RMR17H111, Access Bio, USA) detects *P. falciparum* and non-falciparum parasites (*P. vivax, P. ovale and P. malariae*). With both RDTs, the presence of a line in the ‘C’ window indicated a valid test. Invalid tests were repeated. A valid uRDT test was determined positive if a line was observed in the *P. falciparum*-line (Pf-line) window, whilst a valid csRDT was determined positive if the Pf-line was present regardless of presence or absence of a line in the Pvom window.

Both RDTs were performed according to the manufacturer’s protocol: briefly, a five µl reconstituted blood sample was added to the RDT cassette, followed by three (csRDT) or four (uRDT) buffer drops. RDTs were read according to the manufacturer’s guidelines; after 20 min for uRDTs and 15 min for csRDTs. RDTs were performed at the Eijkman Institute by an operator who was blinded to the reference test results. Presence of a line on a RDT was confirmed by two independent operators. A third operator was consulted in case of uncertainty, and the test was declared positive if two operators agreed.

### Reference standard: composite molecular test

Molecular testing was carried out on DNA extracted (Chelex method) in 2016–2017 from DBS samples. Briefly, 6 mm filter paper punches were incubated on 0.5% saponin overnight, before centrifugation and discard of supernatant. Following rigorous PBS washing, the DBS was heated in 20% Chelex 100-Ion Exchanger for 10 min (Biorad Laboratories, Hercules, CA) at 100 °C, and the remaining supernatant stored at − 20 °C [17].

LAMP and qPCR results formed a composite molecular reference. Discordant results underwent nested PCR testing as the gold standard, which was run in triplicate and results were determined positive if a single replicate was positive by nPCR. These composite molecular tests were used as the reference standard (further described by Ahmed et al. [17]).

Nested PCR was performed on LAMP-positive/qPCR negative samples with primers and cycling conditions described in Singh et al. [23]. The nPCR was used as the gold standard whereby a single positive result determined the sample as positive for *P. falciparum.* The LOD of this assay is ~ 6 parasites/μl [23].

LAMP assays were carried out using the Loopamp™ Malaria Pan Detection Kit (Eiken Chemical Company, Japan). Briefly, the LAMP reagents were reconstituted with 15 μl water/tube and left to stand for 2 min, before adding 15 μl of extracted DNA. Samples were incubated for 40 min at 65 °C and then 5 min at 80 °C. Results were interpreted under a UV light, where a green colour change indicated a positive result.

For qPCR, reactions contained, 2 μl DNA, QuantiFast Pathogen PCR Mix (Qiagen™, Hilden, Germany) and primers and probes published by Kamau et al. for *P. falciparum* and *P. vivax* identification [24], whilst *P. ovale* and *P. malariae* primers and probes are published by Shokoples et al. see Table 1 [25]. Thermocycling conditions (38 cycles) were as follows: 10 min at 95 °C, 15 s for 95 °C and 60 s for 60 °C. Fluorescence was acquired using the Rotor-Gene Q 5plex HRM Platform (Qiagen™, Hilden, Germany) and cycle threshold (Ct) values were calculated using the Rotorgene Q series software version 1.7 (Qiagen Inc, Valencia, CA, USA). The LOD of this LAMP and qPCR assays is 2–5 parasite/μl.

**Table 1:** Working concentrations and sources are provided for the qPCR primers and probes. Table adapted from Ahmed et al. [17]

	Sequence 5′–3′	Concentration (nM)	Source
Pan reverse [25]	AACCCAAAGACTTTGATTTCTCATAA	200	Eurofins
MAL FP [25]	CCGACTAGGTGTTGGATGATAGAGTAAA	50	Eurofins
MAL probe [25]	ATTO700-CTATCTAAAAGAAACACTCAT-MGBEDQ	80	Eurogentec
OVA FP [25]	CCGACTAGGTTTTGGATGAAAGATTTTT	50	Eurofins
OVA Probe [25]	Cy5-CGAAAGGAATTTTCTTATT-MGBEDQ	80	Eurogentec
FAL FP [24]	ATTGCTTTTGAGAGGTTTTGTTACTTT	400	Eurofins
FAL RP [24]	GCTGTAGTATTCAAACACAATGAACTCAA	400	Eurofins
FAL probe [24]	FAM-CATAACAGACGGGTAGTCAT-MGBQ	200	Thermo
VIV FP [24]	GCAACGCTTCTAGCTTAATCCAC	400	Eurofins
VIV RP [24]	CAAGCCGAAGCAAAGAAAGTCC	400	Eurofins
VIV probe [24]	VIC-ACTTTGTGCGCATTTTGCTA-MGBQ	200	Thermo

All molecular testing was carried out by independent operators blinded to clinical information. The LAMP, qPCR and nPCR were carried out a year prior to testing with the uRDT and csRDT. LAMP, csRDT and uRDT tests were carried out at the Eijkman Institute, Jakarta, Indonesia and qPCR testing was subsequently carried out at the Liverpool School of Tropical Medicine, UK.

Positive DNA controls for each species (provided by the Malaria Reference Laboratory, Public Health England) and negative controls were included in every molecular assay run. All assays were carried out by independent operators blinded to clinical information. Index testing was also carried out without knowledge of the reference standard results.

### Sample size

The sample size was calculated using G*Power software v3.1.9.2 (University of Dusseldorf, Germany). Estimating a proportion of 0.35 discordant results between the two RDTs (based on previously published data in similar transmission setting [26]), a sample size of 120 positive samples was estimated to provide sensitivity with 80% statistical power and 99% confidence intervals. Studies have shown both uRDT and csRDT have similarly high specificity (> 96%) [18, 19, 26]. Therefore, the sample size was estimated to be able to detect differences only in sensitivity. The final sample size (n = 270) used for this analysis were those samples with sufficient volumes of both plasma and pRBCs remaining for RDT testing. Of these, 158 were *P. falciparum* positive and 112 negatives by the composite molecular reference test.

### Statistical analysis

Diagnostic accuracy was estimated by calculating the total number of true positives (TP), false positives (FP), true negatives (TN), false negatives (FN), sensitivity and specificity (%), positive predictive value (PPV) and negative predictive value (NPV). PPV and NPV calculated according to prevalence within the sample collection. The reference test method to get the TP, FP, TN and FN was the composite molecular test results (LAMP, qPCR and nPCR). Agreement between either RDT and the composite reference test was calculated by determining the kappa value (*k*). Kappa values < 0.2 were considered a poor agreement, 0.21–0.40 fair, 0.41–0.6 moderate, 0.61–0.8 very good [18]. The diagnostic odds ratio (DOR) was calculated as the ratio of the odds of positivity in those with malaria (defined by molecular composite reference test) relative to the odds of positivity in those without malaria). The Ct values were used as a proxy of parasite densities. The significance of the difference observed between the uRDT and csRDT R^2^ correlation coefficients was assessed using a Fisher r-to-z transformation. The analysis was conducted using SPSS (version 24.0, Armonk, New York) and an online calculator [27, 28] which uses the efficient-score method to calculate 95% confidence intervals.

### Ethical approval

Ethical clearance was obtained from the Liverpool School of Tropical Medicine and the Eijkman Institute for Molecular Biology, Jakarta, Indonesia. Written informed consent was obtained.

## Results

**Fig. 1:**
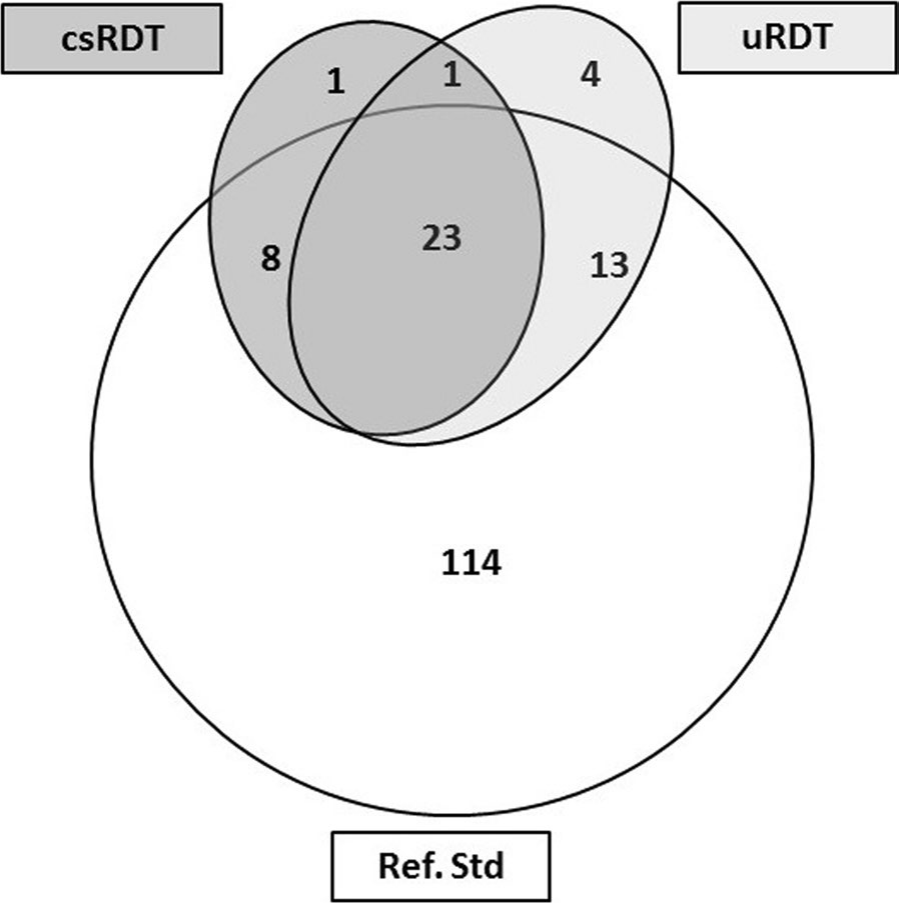
Venn diagram showing the number of *P. falciparum* positive samples detected by each test. Positivity by uRDT, csRDT and the composite molecular reference test (Ref.Std) in 270 samples from asymptomatic pregnant women. Numbers within the circles (n) indicate the number of true positives detected by that test

In total, 270 samples were screened. These included 158 *P. falciparum* positive samples (including non-falciparum *Plasmodium* co-infections) and 112 negative samples (by molecular composite). Only 23 positive samples were detected by either uRDT and csRDT (Fig. 1).

### Agreement with reference test

The level of agreement of either RDT with the reference test was poor: uRDT; kappa value = 0.15 (95% CI 0.09–0.21), csRDT; kappa = 0.16 (0.09–0.23) (Table 2). The uRDT detected 31 (19.6%) infections confirmed by the reference test, of which 23 (74.0%) were also detected by the csRDT (Fig. 1). The uRDT missed 13 (36%) infections that were detected by the csRDT and confirmed by the reference test. The uRDT detected eight infections that were not detected by csRDT (Fig. 1).

**Table 2:** Detection of *P. falciparum* and diagnostic performance of the csRDT and the uRDT

	n	TP	FP	TN	FN	Sensitivity (%)		Specificity %		PPV (%)		NPV (%)		DOR		Agreement (κ)	
Detection of *P. falciparum* including non-*falciparum* species																	
uRDT	270	31	2	110	127	19.6	(13.9–26.8)	98.2	(93.1–99.7)	93.9	(78.4–98.9)	46.4	(40.0–53.0)	13.4	(3.1–57.4)	0.15	(0.09–0.21)
csRDT Pf-line	270	36	5	107	122	22.8	(16.7–30.3)	95.5	(89.4–98.3)	87.8	(73–95.4)	46.7	(79.8–88.8)	6.3	(2.4–16.7)	0.16	(0.09–0.23)
Detection of *P. falciparum* excluding non-*falciparum* species																	
uRDT	242	31	2	82	127	19.6	(13.9–26.8)	97.6	(90.9–99.6)	93.9	(78.4–98.9)	39.2	(32.6–46.2)	10	(2.3–42.9)	0.13	(0.07–0.13)
csRDT Pf-line	242	36	2	82	122	22.8	(16.7–30.3)	97.6	(90.9–99.6)	94.7	(80.9–99.1)	40.2	(33.5–47.3)	12.1	(2.8–51.6)	0.15	(0.09–0.15)

*n* number of samples, *TP* True Positive, *FP* False positive, *TN* True Negative, *FN* False Negative, *Sens* sensitivity, *Spec* specificity, *lowCI* Lower 95% confidence Interval, *UpperCI* Upper 95% confidence interval, *PPV* Positive Predictive Value, *NPV* Negative Predictive Value, *DOR* Diagnostics odds ratio, *k* Kappa value

### Diagnostic performance

The sensitivities compared with the reference test were 19.6%, (95% CI 13.9–26.8) for the uRDT and 22.8%, (16.7–30.3) for the csRDT. Both tests showed high specificity (> 95%). The uRDT DOR was 13.4 (3.1–57.4) and the csRDT 6.3 (2.4–16.7). (Table 2) When comparing the overall performance of the uRDT and csRDT, the difference in proportions detected was non-significantly different (p = 0.169).

### Parasite density distribution

**Fig. 2:**
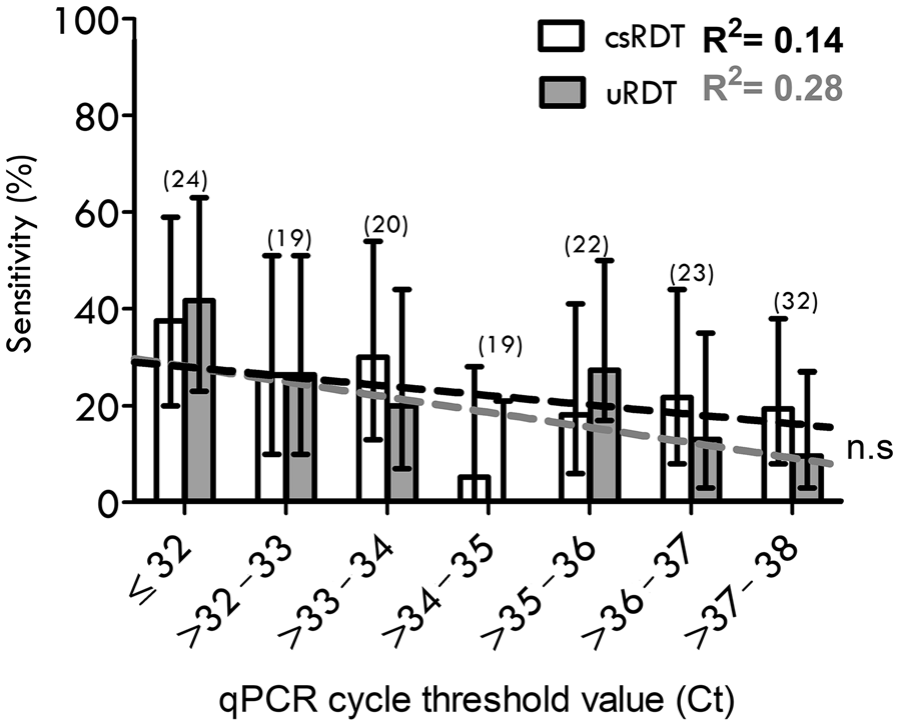
Diagnostic performance stratified by qPCR Ct values. The bar chart shows the sensitivity of each test: uRDT (grey) and csRDT (white bar and black trendline) stratified by qPCR cycle-threshold (Ct) values. The number of qPCR- positive samples in each Ct category is given in brackets (n). Trendlines (dashed) are derived from the rolling mean sensitivity calculated for every 10 observations. R squared coefficient values are displayed (uRDT; grey text, csRDT; black text) and difference between R^2^ values is non-significant (n.s, p = 0.3 one-tailed t-test). (28) Error bars represent upper and lower 95% CI

The qPCR Ct value was used as a proxy for parasite density. Overall, 36% of qPCR positives fell between Ct 33–35 (reflecting an estimated 1–10 parasites/µl), and nearing the reported uRDT LOD of 3.13 p/μl [19], and 25% fell below Ct 30, the estimated LOD of the csRDT LOD (~ 100 parasites/μl) [19]. There was no evidence that the relative performance of the RDTs was modified by CT value (p = 0.3) (Fig. 2).

### Exclusion of co-infections

Considering the uRDT only detects *P. falciparum* infections, the effects of co-infections with *P. vivax* were also assessed. When *P. falciparum*-negative/*P. vivax*-positive samples were excluded from the data set, the number of false positives detected only by the csRDT was reduced (from 5 to 3), thus increasing the specificity and PPV. This resulted in a near doubling of the csRDT DOR (12.1; 2.8–51.6), while the performance of the uRDT remained relatively unchanged. Again, the difference in proportions detected by each test was not different from each other (n = 238, p = 0.4, Table 2).

## Discussion

This study shows that in a moderate transmission setting in Indonesia, amongst asymptomatic pregnant women, the uRDT performed similarly to the csRDTs, which is a widely used test in the malaria in pregnancy control programme in Indonesia.

These results are supported by a study in Colombia, which also used stored samples from pregnant women. Although they note a trend of increased sensitivity by the uRDT, it was not found to be significantly different from the Standard Bioline RDT (sdRDT) [20]. A Tanzanian study in febrile children and adult outpatients also found no difference between uRDT and sdRDT performance, although febrile patients tend to have higher parasitaemia [29]. The results here differ from other studies conducted in non-pregnant populations that showed a significant increase in sensitivity of the uRDT compared to current RDTs. [19, 26, 30, 31]. The most apparent difference between the conflicting results is the target population (pregnant vs non-pregnant) and the storage conditions. It would be useful to screen pregnant populations in the same settings where differences in RDT performance were observed. One such study has commenced in Papua New Guinea (personal communications with Dr Leanne Robinson).

The uRDT is an antigen capture test specific to the *P. falciparum* HRP2 antigen, whereas the csRDT captures pLDH specific to *P. vivax, P. ovale* and *P. malariae* (indicated by the VOM window), in addition to PfHRP2 (*Pf* window). Ahmed et al. showed that amongst pregnant women in Indonesia, when using a different CareStart RDT that captures both pan-LDH and PfHRP2, reading the PfHRP2 window alone was less sensitive than using in combination with the pLDH results [18]. These findings and the low sensitivity of both RDTs demonstrated here, may point towards low HRP2 concentrations that fall below the limit of either RDT (uRDT LOD = 80 pg/ml, csRDT = 200 pg/ml). Low concentrations of HRP2 could be due to low parasitaemia, degradation of the protein during storage or deletions in the gene for this protein.

The sample set included a total of 8 samples that were *P. falciparum*-positive by the FirstResponse RDT performed during the trial (2014–2016), 6 of which were also positive by microscopy. All of these samples were also positive using the uRDT and CareStart in this study. This suggests minimal degradation over the storage period (1–3 years).

The non-linear relationship between HRP2 concentration/persistence and parasite density [19, 26], complicates analyses between RDT positivity and parasitaemia. Although no trend in sensitivity was observed with increasing Ct values (indicative of parasitaemia), however, most samples fell above Ct 30 indicating very low parasitaemias. Finally, HRP2 deletions have been reported in *P. falciparum* from several countries in South America, Africa and also India [32–34], but not yet in SE. Asia. As yet, no full deletions (only polymorphisms [35]) of HRP2 have been reported in Indonesia, although this is an area for further consideration. HRP2 mutations have been implicated in the poor sensitivity of RDTs [33]. Early laboratory analyses of the uRDT using cultured parasites with HRP mutations demonstrated that the uRDT performance varies considerably depending on mutations of the HRP protein [19]. Consequently, investigations around HRP2 polymorphisms in this sample set are underway.

A limitation of this study is the use of different sample types used for the molecular and RDT testing. Molecular testing used stored DNA extracted from DBS, whereas RDTs were performed using pRBC and plasma samples reconstituted to an artificial HCT of 30%. Potential degradation of HRP2 in the stored samples could contribute to poor sensitivity of both RDTs since RDT testing was carried out 1 year after molecular testing, although positive samples remained positive over this time. The sensitivity of the molecular testing could also be enhanced through targeting higher copy genes [36] or RNA [37], or by using improved extraction methods [38]. The performance of both RDTs is anticipated to improve using fresh whole blood at point of contact, i.e. for the intended user scenario. However, the low sensitivity observed here is similar to reported sensitivities using a range of alternative RDTs in pregnant women in Indonesia. [18] The important finding here is that the uRDT performed similarly to the csRDT.

## Conclusion

The diagnostic performance of the uRDT was assessed in a diagnostically challenging population: asymptomatic, pregnant women in a moderate transmission setting in Indonesia. This is the first study testing the uRDT in pregnant women in Asia and will build evidence to guide policy around the implementation of this test in these populations. In comparison to the composite molecular reference tests, both the uRDT and csRDT showed low sensitivity. Given the negative outcomes associated with malaria in pregnancy, it is crucial to detect and treat these infections rapidly. Further work is needed to assess the diagnostic performance of the uRDT in pregnant women before consideration of this test for implementation where csRDT are already being used- particularly in asymptomatic pregnant women in moderate transmission settings.

## Data Availability

The datasets used and/or analysed during the current study are available from the corresponding author on reasonable request.

## Abbreviations

csRDT: CareStart Rapid Diagnostic Test
DBS: dried blood spot
DNA: deoxyribonucleic acid
DOR: diagnostic odds ratio
EDTA: ethylenediaminetetraacetic acid
FN: false negatives
FP: false positives
NAATs: nucleic acid amplification tests
HCT: haematocrit
HRP: histidine rich protein
LAMP: loop-mediated Isothermal Amplification
nPCR: nested polymerase chain reaction
NPV: negative predictive value
PPV: positive predictive value
qPCR: quantitative polymerase chain reaction
RBC: red blood cell
RDT: rapid diagnostic test
RNA: ribonucleic acid
uRDT: ultra-sensitive rapid diagnostic test
sdRDT: standard bioline rapid diagnostic test
STOPMIP: intermittent screening and treatment or intermittent preventing therapy for the control of malaria in pregnancy
TN: true negatives
TP: true positives
